# Method validation of circulating tumour cell enumeration at low cell counts

**DOI:** 10.1186/1471-2407-13-415

**Published:** 2013-09-11

**Authors:** Jeffrey Cummings, Karen Morris, Cong Zhou, Robert Sloane, Matt Lancashire, Daniel Morris, Stephen Bramley, Matt Krebs, Leila Khoja, Caroline Dive

**Affiliations:** 1 Clinical and Experimental Pharmacology Group, Paterson Institute for Cancer Research, Manchester Cancer Research Centre, University of Manchester, Manchester M20 4BX, UK

**Keywords:** Circulating tumour cells, Predictive biomarker, Enumeration, Low cell counts, Method validation, Statistical analysis

## Abstract

**Background:**

Circulating tumour cells (CTC) are receiving increasing attention as prognostic, predictive and pharmacodynamic biomarkers in cancer patients. However, their clinical significance can be dependent on an accurate determination of CTC around cut-off values at low cell counts (<10 cells/7.5 ml). Consequently, we have conducted method validation of the CellSearch™ system focusing on clinical samples containing CTC in the cut-off region.

**Methods:**

Analytical accuracy was first assessed employing quality controls (QC) and spiked healthy volunteer blood specimens. Results were analysed by β-expectation tolerance intervals (BETI). Inter-operator error (6 different readers) was then characterised in 38 different patient samples, 68% of which had ≤5 CTC and data were analysed by β-content γ-confidence tolerance intervals (BCTI).

**Results:**

Results from QCs and spiked blood confirmed a 3-4-fold higher degree of imprecision at the low (48 cells, BETI = + 0.288/-0.345, β = 95%) compared to the high QC (987 cells, BETI = +0.065/-0.140, β = 95%). However, when data for individual analysts were interrogated characteristic systematic errors were detected. In the analysis of patient samples again individual analysts introduced a highly specific error into the interpretation of CTC images, which correlated to the level of training and experience. When readers were selected based on BETI and BCTI results, the high level of between-operator error (up to 170%) observed at CTC of ≤ 5 was reduced to < 30%.

**Conclusions:**

Inter-operator variability in enumeration of CTC at low cell counts can be considerable, but is also potentially avoidable by following simple guidance steps.

## Background

Detection, enumeration and characterisation of circulating tumour cells (CTC) as a potential biomarker currently represents one of the most actively pursued areas in translational cancer research [1]. CTC are believed to act as the ‘seeds’ for the establishment of metastatic disease, and also a mechanism to re-populate the primary tumour, and their presence has been shown to correlate to both progression free survival and overall survival [2-5]. In these studies, a discrete cut-off point was discriminated at extremely low numbers of CTC above which poorer prognosis was observed: ≥ 5 in 7.5 ml blood for metastatic breast cancer, metastatic castration resistant prostate cancer and non-small cell lung cancer; ≥ 3 for metastatic colorectal cancer; ≥ 2 in melanoma and ≥ 1 in neuroendocrine tumours [2-8]. In addition, CTC have been shown to be predictive of response to both chemotherapy and targeted agents in post-treatment samples and to act as a pharmacodynamic biomarker [2,9-12].

Isolation of rare cells (1 CTC in 10^8^ leucocytes) presents considerable technological challenges requiring a robust analytical technique and a number of different approaches have been developed based on the physical and biological properties of CTC [1,13]. To date, the CellSearch™ system (Veridex, Raritan, NJ, USA) remains the only platform that is cleared by a regulatory authority (the FDA in the USA), as an aid in the monitoring of patients with metastatic breast, colorectal and prostate cancer http://www.accessdata.fda.gov/cdrh_docs/pdf7/K073338.pdf. The system consists of two major instrumental components: the AutoPrep station for the fully-automated immunomagnetic isolation of cells from whole blood and an Analyser for the semi-automated identification of CTC based on 4-channel fluorescence microscopy [14]. Although, the subject of method validation studies in the past, these have tended to focus on either precision of quality control samples or between laboratory concordance (reproducibility) [15,16]. Only limited studies have been performed evaluating the analytical accuracy of the technique - determination of the true value for CTC in the patient sample [17]. Nonetheless, CellSearch remains the benchmark against which all new technologies should be assessed [18].

Assays employed as a prognostic or predictive biomarker require a credible level of analytical validation [19,20] and in the case of CTC that should include a demonstration that the technique is accurate and reproducible at the cut-off level [18,21]. Therefore, in the present study method validation of the CellSearch system was conducted focusing on the analysis of patient samples containing low cell counts, in the region of the published clinically relevant cut-off points. To address the issue of analytical accuracy statistical approaches to the interpretation of data including β-expectation tolerance intervals (BETI) were employed [22]. In addition, a major goal was to achieve a reduction in inter-operator variability and this aspect utilised a modification of incurred sample reproducibility (ISR) and β-content γ-confidence tolerance intervals (BCTI) [23].

## Methods

### Patients and blood sample collection

Blood samples (7.5 ml) for CTC enumeration were collected from a total of 38 different lung, prostate, melanoma and colorectal patients receiving standard of care chemotherapy at the Christie Hospital, Manchester and entered into a number of experimental medicine studies being conducted at the Paterson Institute for Cancer Research. Written informed consent was obtained from all subjects and the studies were ethically approved by the Tameside and Glossop Research Ethics Committee (Manchester, UK) and the Declaration of Helsinki Principles was followed. Samples were harvested into CellSave tubes (Veridex, Raritan, NJ, USA), containing EDTA and a cellular preservative and maintained at room temperature for no longer than 72 hours prior to analysis. Blood was also collected from healthy volunteers for recovery experiments according to a local ethics committee approved protocol.

### CTC enumeration by CellSearch

CTC were essentially enumerated as previously described in detail [7,24,25]. In brief, blood was diluted, centrifuged and incubated with ferrofluid particles coated with anti-EpCAM antibodies utilising the CellTracks AutoPrep station (Veridex). After immunomagnetic enrichment, ferrofluid-captured cells were permeabilised and fluorescently labelled using phycoerythrin-conjugated anti-cytokeratin antibodies (pan-keratin antibody C-11) to identify epithelial cells and allophycocyanin conjugated anti-CD45 antibody to identify and discount leucocytes. 4-6-Diamidino-2-phenylindole (DAPI) was incorporated to identify cell nuclei. Upon repeated magnetic separation, the fluorescently labelled cells were oriented to the surface of the (MagNest™) cartridge for interrogation using the CellTracks Analyser II (Veridex). Image frames covering the entire surface of the cartridge were captured by the software and a gallery of objects meeting pre-determined criteria presented to the analyst to confirm or otherwise the presence of CTC. Image galleries were assessed by the operator without prior knowledge of patient data. A CTC is defined as a nucleated cell staining positively for cytokeratin and negatively for CD45 and results are reported as CTC number per 7.5 ml of blood.

### Experimental studies

To establish the level of analytical accuracy achievable by the CellSearch system, the quality control (QC) reagents provided by Veridex were utilised. These are certified to contain a specified range of human tumour cells (SK-BR3 cells), at a high and low cell count, and are integral to the quality control procedures of the system. The statistical evaluation of analytical accuracy employed β-expectation tolerance intervals (see below). QC data obtained over a 3 month period during the analysis of 27 different batches of patient samples were collated for statistical evaluation. Variables investigated included the influence of two different CellSearch systems and 3 different operators.

In a second statistical evaluation of analytical accuracy by BETI, healthy donor blood was spiked with a known number of human tumour cells according to the following protocol. Approximately 30 ml of normal donor blood was collected into a CellSave tube. Growing cultures of either SW620 or H1048 cells were trypsinised and re-suspended in 1 ml phosphate buffered saline (PBS) and counted. Cells were re-suspended to a final concentration of either 3 or 25 in 100 μl (i.e. 30 or 250/ml) in PBS. 100 μl of cell suspension was then added to 3 empty CTC isolation tubes followed by 7.5 ml of normal donor blood. A control sample of 7.5 ml of normal donor blood with no spiked cells was also included. The number of cells spiked to each tube was unknown to the 6 different operators who then enumerated the cell numbers by CellSearch.

The final experiment involved the interrogation by 6 different operators of the same image galleries obtained from the analysis of 38 different patient samples. Analysts were selected on the basis of varying levels of training and experience. In the statistical evaluation of the resultant data a two-sided β-content γ-confidence tolerance interval was employed (see below).

### Mathematical calculations and statistical analysis

Calculation of BETI was preformed utilising MATLAB (Version R2009a, MathWorks, Natick, MA, United States) as described previously [26]. Tolerance intervals were calculated at β = 67%, 80% and 95%. A plot of BETI (y-axis) against the nominal concentration of the QCs or spike (x-axis) is referred to as the ‘accuracy profile’ and is used extensively throughout this report.

Evaluation of ISR utilised BCTI for statistical analysis of data. This yields an upper and lower interval where a specified (β) proportion of measurements will lie with a specified (γ) level of confidence and was calculated as previously reported [23]. In our adaptation of this methodology, where normally a single operator assays the same samples twice (or more), data from a pair of operators who assayed the same samples a single time were substituted into the calculations in order to characterise the relative error introduced by each. Here,

$$Y_{i}^{O}$$

= the original measurements (i.e. analyst 1).

$$Y_{i}^{R}$$

= the repeat measurements (i.e. analyst 2).

$$\Delta_{i}=\log\left(Y_{i}^{R}\right)-\log\left(Y_{i}^{O}\right)$$

$$\bar{\Delta \;}=\frac{1}{N\;}\;\sum_{i=1}^{N}\Delta_{i}$$

$${\hat{\sigma }}_{\Delta }^{2}=\frac{1}{N-1}\;\sum_{i=1}^{N}{\left(\Delta i-\bar{\Delta }\right)}^{2}$$

Where, *Δ*_*i*_ is the difference between original and repeat measurements in log transformed concentrations, *N* is the number of patient samples, $\bar{\Delta }$ is the mean of the differences between the log transformed concentrations and ${\hat{\sigma }}_{\Delta }^{2}$ is the variance of the differences between the log transformed concentrations. The two tailed β-content γ-confidence tolerance interval is therefore defined as:

$$\bar{\Delta }\pm Z_{\left(1+\beta \right)/2}\sqrt{1+N^{-1}}\;\sqrt{\left(N-1\right)\;{\hat{\sigma }}_{\Delta }^{2}/x_{N-1,1-\gamma }^{2}}$$

*Z*_(1 + *β*)/2_ is the upper (1 + *β*)/2 quantile of the standard distribution and $x_{N-1,1-\gamma }^{2}$ is the lower γ quantile of the chi-squared distribution (N-1 degrees of freedom). Calculation of BCTI was performed utilising MATLAB (as above) at β = 67% and 95% [26]. A plot of BCTI (y-axis) against the operator pair (x-axis) represents a modified form of the ‘accuracy profile’. All code developed in MATLAB was validated against previously published data sets as reported previously [26].

## Results

### Analytical accuracy of the CellSearch system characterised by β-expectation tolerance intervals

**Figure 1:**
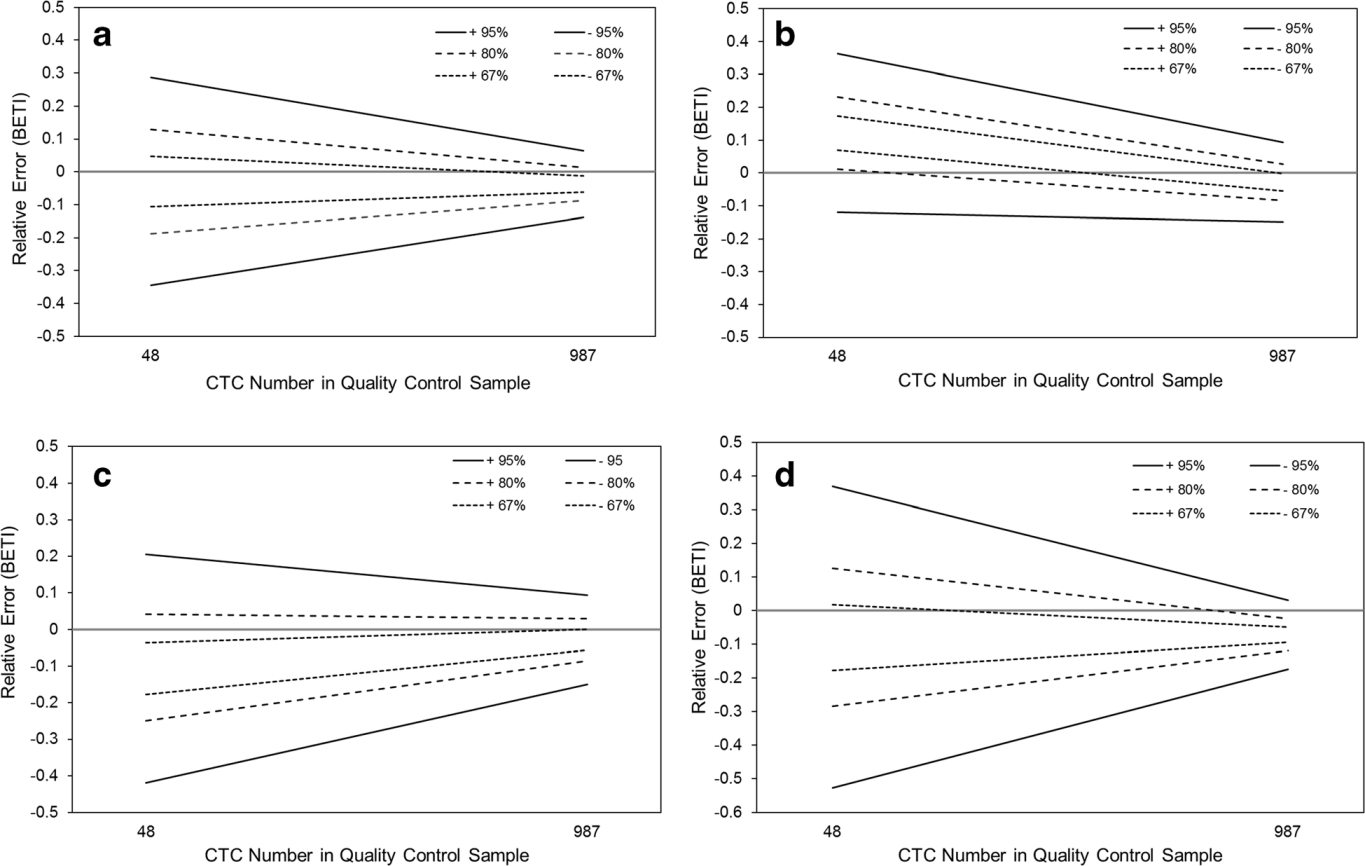
**Determination of analytical accuracy in CTC enumeration utilising BETI and QC samples.** Certified QC samples containing SK-BR3 human tumour cells spiked at high and low numbers were assayed by a pool of analysts over a 3 month period in order to construct tolerance intervals (±) at β = 95%, 80% and 67%. Combined tolerance intervals for all analysts **(a)** versus tolerance intervals for a single analyst: **(b)**, **(c)** and **(d)**.

Figure 1A contains the accuracy profiles and BETI generated from the QC data obtained over a 3 month period during the analysis of 27 different batches of patient samples. In keeping with previously published data [15,27], the error associated with the analysis of CTC at lower numbers was 2–3 fold greater than at higher numbers. There appeared to be little evidence of bias (systematic error) at either high or low CTC numbers, where the tolerance intervals at β = 67, 80 and 95% were symmetrically centred about the mid-point of the certified range for the QCs. In absolute terms, the tolerance intervals rarely exceeded a margin of 30% (even at 95% probability), the recommended benchmark for a biomarker assay in the fit-for-purpose approach to method validation [17].

Nonetheless, when the equivalent data were plotted for each operator involved in the analysis (see Figure 1B-D), striking differences in the resultant accuracy profiles emerged (P < 0.05, ANOVA). Analyst 1 introduced a positive bias in the determination of the low QC which was significantly different from the performance of the other two analysts (Newman-Keuls multiple comparison test) while Analyst 2 introduced a negative bias in the low QC. A large degree of imprecision (random error) was evident in the low QC data attributed to Analyst 3 coupled to a small negative bias in the high QC. Identification of such discrete analytical errors allows for the possibility of their correction, demonstrating the potential power of the BETI approach to method validation. No significant differences in QC values were recorded when the results obtained from the two separate CellSearch systems were compared (Student’s t test).

Six different operators independently interrogated the image galleries produced by the analysis of volunteer blood samples spiked with low numbers of tumour cells (3 and 25, n = 3). Here, BETI for the samples spiked with 25 cells was +0.562 and −0.546 at β = 95%, total error was 24.5% and average recovery 101% ± 24% coefficient of variation (CV), consistent with a large degree of random error but absence of systematic error and in keeping with previous studies [16]. Due to the relatively small number of specimens in this study, it was not possible to discriminate the individual contribution of each analyst towards the overall level of error. BETI for the samples spiked with 3 cells was +0.486 and −0.264 at β = 95%, total error was 28.3% and average recovery was 90% ± 9.6%.

### Incurred sample reproducibility of the CellSearch system characterised by β-content γ-confidence tolerance intervals

**Figure 2:**
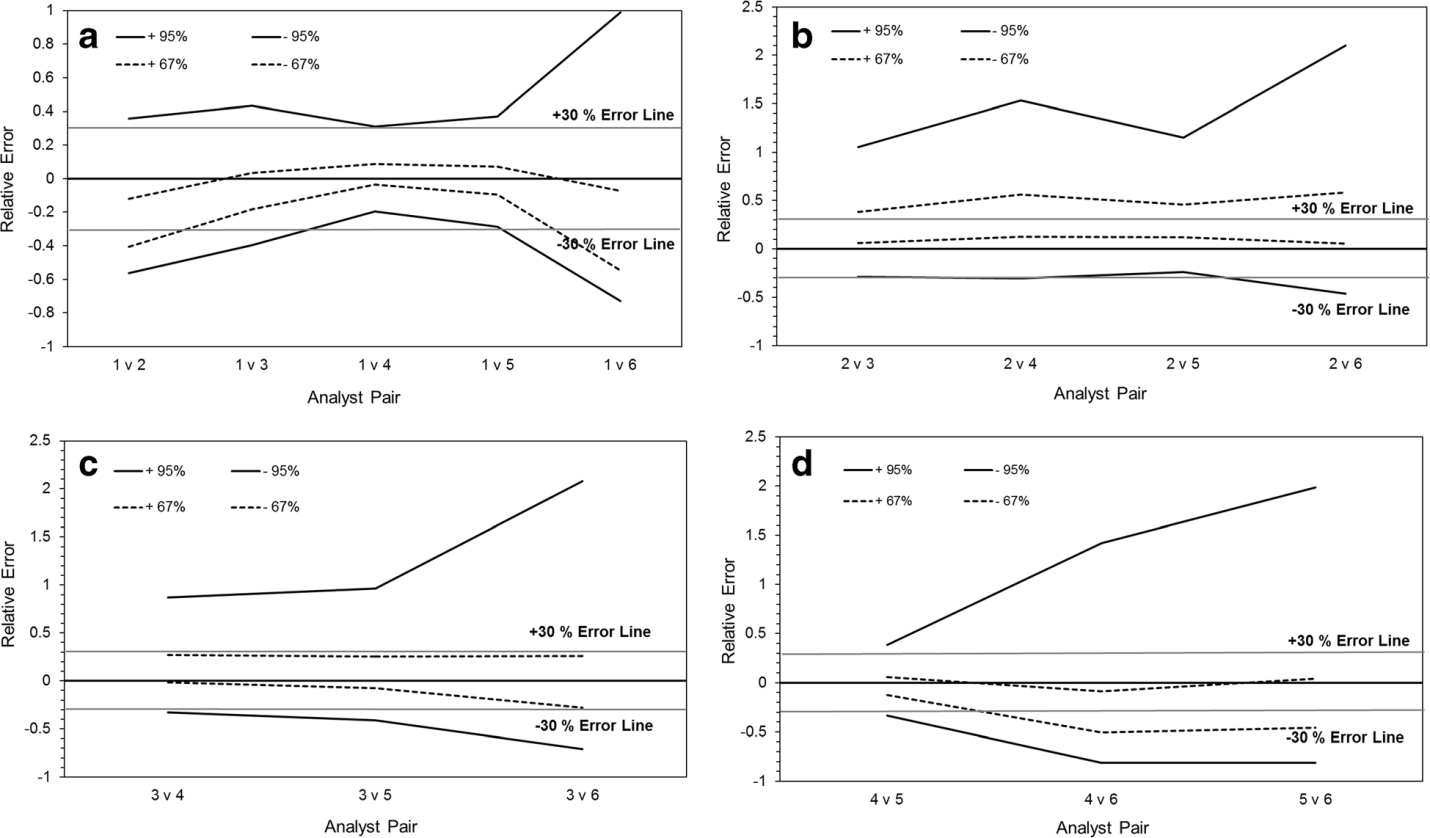
**Characterisation of inter-operator variability in CTC enumeration at low cell counts utilising BCTI and patient samples.** Image galleries generated from the analysis of different cancer patient blood samples, 68% of which had ≤5 CTC, were enumerated by a pool of operators. Results were analysed by a modification of incurred sample reproducibility where the counts obtained by a pair of operators who interrogated the same samples were substituted into the calculations. Tolerance intervals (±) were constructed at β = 95% and 67% and the ± 30% error line is shown for reference. **(a)**, Analyst 1 versus analysts 2–6; **(b)**, analyst 2 versus analysts 3–6; **(c)** analyst 3 versus 4–6 and **(d)** analyst 4 versus 5 and 6 and analyst 5 versus 6.

The nature and extent of inter-operator error in CTC enumeration by CellSearch was investigated through ISR and applying this concept to different pairings of analysts (Figure 2A-D). Of all patient samples analysed and interrogated in this evaluation a total of 68% had nominal CTC values of ≤5. Figure 2A contains the ‘modified’ accuracy profiles and BCTI for a pairing of all 6 operators, each of whom was selected on the basis of differing levels of training and experience. Analyst 1 was the benchmark, the most highly trained and experienced member of the group. The level of error recorded between the different pairings was comparable and in certain cases (e.g. analyst pair 1 and 4) less than that observed in the analysis of the QC samples (see Figure 1), a phenomenon previously noted in biomarker method validation of 2 cell death ELISA assays [26]. The magnitude of error between pairs of operators followed very closely their level of training and experience. Analysts 4 and 5 underwent professional training at the Veridex European Centre and were highly experienced whereas Analyst 6 had only recently received in-house training, while Analysts 2 and 3 were intermediate in experience.

When Analyst 2 was compared against the other operators, there was a considerable increase in the tolerance intervals recorded (approximately 2-fold) and the introduction of a strong positive bias (Figure 2B). Likewise, in the case of Analyst 3, who was intermediate in experience, there was also a large increase in the level of random error but without any notable bias (Figure 2C). Analyst 6, the most in-experienced operator, was consistently associated with a much greater level of error than any other analyst (see Figures 2A-D). Apart from Analyst 1, the benchmark operator, the two other experienced analysts - 4 and 5 - appeared to be able to function within or close to the 30% margin of error recommended as acceptable for biomarker assays [17] (Figure 2D).

### Amelioration of the inter-operator error associated with the enumeration of low CTC utilising BETI and BCTI

**Figure 3:**
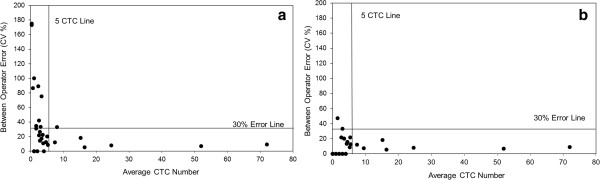
**Amelioration of inter-operator variability in CTC enumeration in patient samples at low cell counts.** Between-operator error was calculated as the coefficient of variation (CV) in the mean cell count obtained by a panel of analysts enumerating CTC in 38 different cancer patient blood samples. **(a)** Inter-operator error for all 6 operators. **(b)** Reduced level of error obtained when only analysts 1, 4 and 5 (see Figure 2) who had demonstrated consistency were included. The 30% error line and 5 CTC line are shown for reference.

Through the application of both certified QCs and patient samples, and employing the statistical procedures of BETI and BCTI in data interpretation, distinct performance characteristics associated with different analysts have been identified. Figure 3A displays the inter-operator error in CTC enumeration at low counts as CVs when data from all 6 analysts were included. The graph illustrates a profile typical of that obtained in previous analogous studies, where there is essentially an exponential increase in error as the cell count approaches zero [16,24,27]. By selecting only analysts who through BETI and BCTI analysis have demonstrated consistency (analysts 1, 4 and 5) the high level of between-operator error observed at CTC of ≤ 5 was significantly reduced and the CV in virtually all cases was less than 30% and in a majority of cases less than 20% (Figure 3B).

## Discussion

The aim of the present study was to improve the accuracy of CTC enumeration by the CellSearch system at low cell counts (<5) utilising the statistical produces of BETI and BCTI. In a seminal paper published by scientists based at Veridex (then called Immunicon Corporation) a statistical model was developed to describe the main sources of error associated with the determination of CTC in human blood [24]. Three major error components were identified: a) sample collection, b) recovery of CTC through immunomagnetic depletion and c) inter-reader variability in the assignment of objects. A stimulus to conduct this study was based on the apparent arbitrary nature of the cut-off point of 5 CTC observed in metastatic breast cancer patients [2,24]. No clear biologic basis could be proffered for such a discrete value [3,8,24,28].

To explain the uncertainties associated with sample collection in CTC enumeration Poisson statistics were incorporated into the error model [24]. Poisson statistics are believed to describe most accurately the effect of counting randomly distributed objects, such as CTC, in a discrete volume [28]. The effect of Poisson statistics on CTC enumeration is illustrated as follows. Where the true number of CTC is 5, the probability of detecting 5 cells in a single sample collected from a patient is relatively small (17.5%). A feature of the Poisson distribution is that the variance is equal to the population mean. Thus, the theoretical CV for a set of measurements carried out on the same samples, based entirely on statistical probabilities, is 44.7% at 5 CTC. A number of previous validation studies, including the present work, have confirmed good agreement between the level of experimental error measured at low CTC and the theoretical level of error dictated by Poisson statistics [16,27].

While it is difficult to control for the uncertainties introduced in the analysis of CTC by Poisson statistics, other than collecting a larger volume or many replicates of the same sample [24,28,29], the other two components in the error model are more amenable to correction. In the case of sample recovery, this component has been demonstrated to exert only a modest effect [16,27], which was confirmed in the present study using spiked healthy volunteer blood samples.

Where there is scope for improvement is in the area of inter-reader variability, which has been proposed as one of the main reasons to explain the arbitrary nature of the cut-off levels observed in clinical trials [3,24,28]. Indeed, it is possible that due to experimental error the true cut-off levels may be even lower than those previously reported [24]. Inter-operator error was also identified as the major contributor to between-laboratory variations observed during an external quality assurance assessment programme [21].

To apply BETI to method validation requires that the true or a certified value of the analyte of interest is known [30]. This limited our evaluation to data derived from the QC samples provided by the vendor (Veridex). BETI is normally associated with bioanalytical techniques, although there are limited examples of its application in cut-off interpretation of ELISA data [31]. To the best of our knowledge, the present report represents the first to apply this approach to method validation of the CellSearch system for CTC analysis. The strength of this procedure is that it informs on analytical accuracy and the quality of result obtained in future measurements, at any operator defined level of probability [30,32]. Results obtained with the QC samples made two significant observations. First, it confirmed that a high level of analytical accuracy was possible, with virtually no bias observed at the high QC coupled to a low level of imprecision. Here the total error was always less than 15%, well within the recommended level for a biomarker technique [33,34]. Second, and most importantly, it identified that at lower CTC, characteristic systematic and random errors could be ascribed to individual analysts.

However, it is also well recognised that QC samples often reflect poorly the analytical behaviour of clinical specimens, especially in the biomarker field [35,36]. In the case of the CellSearch system, the QCs comprised breast cancer cells reconstituted into a non-biologic matrix. Therefore, the main focus of the present paper revolved around the analysis of cancer patient specimens containing low numbers of CTC (<5). To identify inter-reader errors the ISR methodology described by Hoffman was adopted utilising BCTI [23]. In our modification of this process we substituted a different operator to analyse the repeat sample. To control against sampling artefacts, a relatively large number of patient specimens were analysed in a number of different assays [23]. Results obtained clearly showed that an individual analyst could introduce a highly specific error into the interpretation of CTC images, analogous to the QC data. However, it was also demonstrated that with training and experience these errors could be significantly reduced.

Increasingly, the regulators in the USA and Europe are placing more stringent requirements on the validation of biomarker assays [36-38]. Hence, validation data will be adjudged in the future, not merely on the basis of technical performance characteristics with QCs [39], but in terms of the quality and significance of the data generated during the analysis of clinical specimens. As the gold standard, the most reliable biomarker data will be derived from multi-centre trials, where the analysis is subject to external proficiency testing schemes and inter-laboratory comparison programmes [18]. This is especially true when data generated by the assay is intended to be used in the stratification of patients into different treatment arms, thus defining the biomarker as integral to the progress of the trial [19,20]. Here, the assay will have to be demonstrated to possess a proven ability to discriminate between different cohorts with a high degree of diagnostic sensitivity and specificity [37,38]. The first clinical study to conduct patient stratification based on a CTC cut-off value is SWOG S0500 (NCT00382018) in women with metastatic breast cancer receiving chemotherapy [18,40]. Other stratification trials employing CTC as a biomarker are likely to follow, such as the ‘CriTiCal Trial’ (Circulating Tumour Cell guided Chemotherapy Trial in Colorectal Cancer) planned in the UK.

## Conclusions

It has been shown that while inter-operator variability in enumeration of CTC at low cell counts can be considerable; it can be ameliorated by following simple guidance steps. First, and perhaps logically, operators should be trained to a high degree and experienced in the field. Second, utilising statistical based techniques, potential analysts should confirm in a training set of images derived from 30–50 patient samples run in 5–10 different assays, that consistency can be achieved against benchmark analysts. Finally, CTC enumeration in patient samples should be conducted on 2 or preferably 3 separate collections of blood, with a different operator analysing each, for two reasons. First by collecting up to 3 samples, one may attenuate the unavoidable uncertainties imposed on CTC enumeration by Poisson statistics, increasing the probability of detecting ≥ 1 (when present) up to 95% [24]. Second, by employing different analysts one can confirm or otherwise the lack of inter-operator variability.

## Abbreviations

CTC: Circulating tumour cells; QC: Quality controls; BETI: β-expectation tolerance intervals; BCTI: β-content γ-confidence tolerance intervals; ISR: Incurred sample reproducibility; PBS: Phosphate buffered saline; CV: Coefficient of variation.

## Competing interests

The authors declare that they have no competing interests.

## Authors’ contributions

JC, KM, CZ and CD were the main authors of the manuscript. CZ developed and validated all programming code utilised in statistical analysis. JC conducted the statistical analysis and interpretation of data. KM, RS, ML, DM and SB performed all the laboratory analysis of samples. MK and LK collected blood samples and clinical data from patients. All authors have read and approved the final version of the manuscript.

## Pre-publication history

The pre-publication history for this paper can be accessed here:

http://www.biomedcentral.com/1471-2407/13/415/prepub
